# Comparison of *β*-Propiolactone and Formalin Inactivation on Antigenicity and Immune Response of West Nile Virus

**DOI:** 10.1155/2015/616898

**Published:** 2015-08-27

**Authors:** Pritom Chowdhury, Rashmee Topno, Siraj A. Khan, Jagadish Mahanta

**Affiliations:** ^1^Arbovirology Group, Entomology and Filariasis Division, Regional Medical Research Centre, ICMR, Northeast Region, Post Box No. 105, Dibrugarh, Assam 786001, India; ^2^Department of Biotechnology, Tocklai Tea Research Institute, TRA, Jorhat, Assam 785008, India

## Abstract

West Nile Virus (WNV) is a pathogenic arbovirus that belongs to genus *Flavivirus* under family Flaviviridae. Till now there are no approved vaccines against WNV for human use. In this study, the effect of two alkylating agents, formaldehyde and *β*-PL, generally used for inactivated vaccine preparation, was assessed on the basis of antigenic and immunogenic potential of the inactivated WNV. Lineage 5 WNV isolates were inactivated by both formalin and *β*-PL treatments. Inactivation was confirmed by repeated passage in BHK-21 cell line and infant mice. Viruses inactivated by both the treatments showed higher antigenicity. Immune response in mice model showed serum anti-WNV antibody titre was moderately higher in formalin inactivated antigen compared to *β*-PL inactivated antigen. However, no significant differences were observed in neutralization antibody titre. In conclusion, we can state that both formaldehyde and *β*-PL inactivation processes were found to be equally efficient for inactivation of WNV. However, they need to be compared with other inactivating agents along with study on cell mediated immune response.

## 1. Introduction

West Nile Virus (WNV) is a pathogenic arbovirus that belongs to genus* Flavivirus*, family Flaviviridae. The natural cycle involves reservoir hosts, namely, wild and domestic birds. Mosquitoes, generally* Culex* species, act as principal vectors and humans act as dead end hosts [1, 2]. Although most of the human WNV infections remain subclinical, febrile illness and neuroinvasive disease develop in ≈20% and <1% of the infected patients, respectively [3]. Initially, the virus was distributed in Africa, Asia, and Europe where it caused infrequent and unpredictable epidemics of mild systemic disease [4]. However, in recent times the virus has spread rapidly to new regions like Romania (1996), United States (1999), and recently Greece and Italy (2010), resulting in hundreds of neurological and fatal cases worldwide [4, 5]. In India, antibodies against WNV were first detected in human sera from Bombay (1952) [6]. Since then, febrile illness in epidemic form and clinically overt encephalitis cases has been observed from southern, central, and western India [7]. In eastern India, WNV infection causing acute encephalitis syndrome was first reported during 2006 from the state of Assam [8]. WNV has emerged in recent decades as significant burden to public health and its subsequent spread throughout the world has depicted it as a reemerging global pathogen [9]. Currently, there are five inactivated and chimeric vaccines licensed for veterinary use [10]. However, no human vaccine is available although several candidate vaccines are in clinical trial [11]. The economical impact of both clinical and subclinical diseases warrants search for and use of efficient vaccines. Formalin and *β*-propiolactone (*β*-PL) are commonly used for inactivation of viruses via chemical reaction with viral capsid proteins and nucleic acids [12]. Both formalin and *β*-PL have been classified by International Agency for Research in Cancer (IARC) under groups 2A and 2B, respectively [13]. But till now there is no epidemiological data relevant to the evaluation of the carcinogenic risk of the alkylating agent on humans. In general, preserving the integrity of the immunological epitopes is important for vaccine efficacy. Here, we have studied and compared the effect of two alkylating agents, formaldehyde and *β*-PL generally used for vaccine preparation [10, 14] on the antigenic and immunogenic properties of the WNV.

## 2. Materials and Methods

### 2.1. Virus Strain and Cell Propagation

A circulating strain of WNV, WNIRGC07, isolated in 2008 from human AES patient (GeneBank ID: HQ246154) from Assam, India, was used. Phylogenetic analysis placed it on lineage 5 of WNV [15]. Four virus passages in infant* Swiss albino* mice and subsequently two passages in baby hamster kidney (BHK-21) cell line were done to increase adaptability and virus titer. The cell line was obtained from National Centre for Cell Science, Pune, India, and maintained in Eagles Minimal Essential Medium (EMEM, Sigma) supplemented with 10% heat inactivated fetal bovine serum, 7.5% sodium bicarbonate (Sigma), and 2 mM L-glutamine. Tissue culture infectious dose 50 (TCID_50_) of the virus was calculated by cytopathic effect (CPE) method as per protocol of Cui et al. [16].

### 2.2. Virus Inactivation

The virus infected culture supernatant was clarified by centrifugation at 10,000 rpm for 1 hr at 4°C. *β*-PL was added to virus suspension at a concentration of 0.1% and kept for 48 hrs at 4°C [17]. Formalin inactivation was done at a concentration of 0.2% and kept for 5 days at 32°C [18]. Both the suspensions were centrifuged at 10,000 rpm for 1 hr at 4°C and the collected supernatant was treated with protamine sulphate (1 *µ*g/mL) and kept at 4°C for 30 min. Subsequently, the suspensions were centrifuged at 5,000 rpm for 20 min and the supernatant was collected and stored at −80°C till further experiment.

### 2.3. Screening for Virus Infectivity

Inactivated virus preparations were tested for residual infectivity by serial passage into BHK-21 cells and by intracranial inoculation of inactivated virus into suckling mice. For residue of any viral nucleic acid, viral RNA was extracted using QIAamp viral RNA mini kit (Qiagen, Germany), from the brains collected from mice surviving 21 days after inoculation. Reverse transcriptase- (RT-) PCR was done by WNV specific NS5 region using the primer sequence forward 5′-GCTCCGCTGTCCCTGTGA-3′ and reverse 5′- CACTCTCCTCCTGCATGGATG-3′ [19].

### 2.4. Haemagglutination (HA) Assay

Inactivated virus titers were determined by HA assay as described by Clarke and Casals [20]. Serial twofold dilutions of inactivated virus in bovine albumin phosphate buffer (0.4% BABS) were prepared and incubated in round bottom plate with 0.4% goose erythrocytes in variable antigen diluents (VAD) of pH 6.2, 6.4, 6.6, and 6.8. After incubation at room temperature for 30 min, the hemagglutination titer, expressed as the reciprocal of the highest dilution producing complete hemagglutination was read.

### 2.5. Inactivated Virus Antigenicity

The antigenicity of inactivated virus preparations was determined by indirect antigen capture ELISA by following standard method [21]. Ninety-six-well microtiter plates (Nunc-MaxiSorp) were coated with 50 *µ*L/well of* Flavivirus* specific monoclonal antibody (HX-2Ab) at a dilution of 1 : 50 in coating buffer. Biotinylated HX-B (courtesy: National Institute of Virology, Pune, India) was used as detector antibody. ELISA reading was taken at 490 OD in triplicate.

### 2.6. In Vitro Microcytotoxicity Assay/Cell Viability Assay

Cell toxicity assay of inactivated virus preparations was evaluated for cell cytotoxicity in BHK-21 cell lines by using 3-(4,5-dimethylthiazol-2-yl)-2,5-diphenyltetrazolium bromide (MTT) Cell Proliferation Kit (Roche). The percentage of cytotoxicity was calculated as [(*A* − *B*)/*A* × 100] where *A* and *B* are the absorbances of control and treated cells, respectively [22].

### 2.7. Mice Immunization

For immunogen preparation standard inactivated virus preparations (*β*-PL and formalin) were mixed with equal volume of Alhydrogel (2% alum, Sigma), incubated at room temperature for 30 min, and stored at 4°C [23]. A total of two groups (*n* = 8 mice each) of 3- to 4-week-old* Swiss albino* mice were immunized subcutaneously with 50 *µ*L of immunogen. Control group was injected with phosphate buffered saline (PBS). Booster injections with same formulation were given on 14 and 28 days after first immunization.

### 2.8. Determination of Immune Response

The anti-WNV IgG antibody response in inoculated mice was determined on serum samples collected at 7 days after inoculation by indirect ELISA by following standard procedure. Briefly, a 96-well microtiter plate (Nunc-MaxiSorp) was coated with mouse brain derived WNV antigen at 1 *μ*g/mL in coating buffer (pH 9.6) and kept overnight at 4°C. The plate was blocked with 1% BSA (Sigma) in PBS (pH 7.4). After washing with PBST, wells were incubated with postimmunized sera in triplicate wells (100 *µ*L/well). A serum of control group mice was also included along with standard anti-JEV polyclonal sera raised with JEV strain, P20778, for cross-reactivity screening. Bound antibodies were detected by HRP-labeled goat anti-mouse IgG (Sigma). ortho-Phenylenediamine dihydrochloride (OPD) (Sigma) as chromogen and hydrogen peroxide (H_2_O_2_) as substrate were used. Colour development was stopped using 1 M H_2_SO_4_ and plates were read at 490 nm by ELISA reader. To determine the end point of antibody titer, absorbance reading ≥ to twice the OD value of negative control serum was considered positive.

To determine neutralization antibody, hyperimmune sera raised against respective viral inactive agent with WNV strain were used. The fourfold diluted sera were mixed with 100 plaque forming units (P.F.U.) of virus strain G22886 and incubated at 37°C for 1 hr. Virus diluents (0.1 mL) mixture was allowed to absorb in preseeded confluent cell monolayer of BHK-21 cell in a 24-well microtiter plate and incubated at 37°C for 1 hr. Then, cells were overlaid with equal amount of 2x MEM and 1.8% carboxymethyl cellulose (CMC) supplemented with 2% FCS. After 60 hrs, medium was discarded and stained with 0.1% amido black staining solution. Neutralizing antibody titers were expressed by 50% of plaque reduction (PRNT_50_).

### 2.9. Statistical Analysis

Results are expressed as mean values for at least three experiments. Comparison between the two groups was made using two sample independent *t*-tests.

## 3. Result

### 3.1. Inactivation of WNV

WNV inactivation was confirmed after repeated passage into infant mice and BHK-21 cell monolayer. No mortality was observed in infant mice inoculated intracranially, whereas infants inoculated with live virus died after 3-4 days of inoculation. CPE was not observed in both the inactivated virus infected cell monolayers. PCR amplicons also showed no traces of viral nucleic acid.

### 3.2. HA Titer and Antigenicity

**Figure 1:**
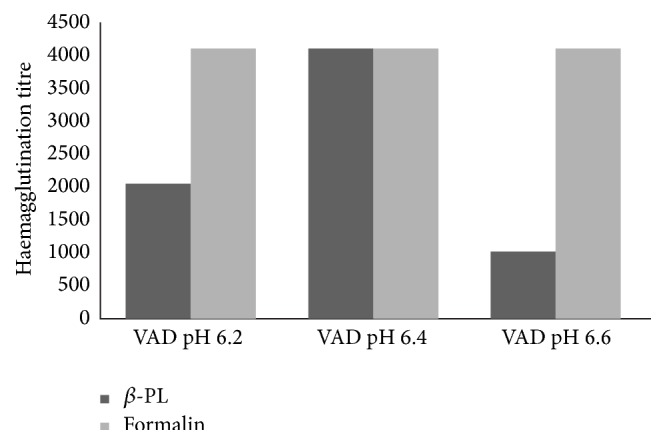
Haemagglutination titre of prepared WNV antigen by two different inactivating agents: *β*-PL and formalin at VAD of pH 6.2, 6.4, and 6.6.

HA assay showed highest titre of 4096 in both the *β*-PL and formalin inactivation procedure. However, in *β*-PL inactivation, antigen titer was found to be dependent on pH values of virus diluent (VAD) where highest titre was obtained at VAD of pH 6.4. However, in antigen prepared with formalin inactivation, uniform high titres were obtained in VADs of pH 6.2, 6.4, and 6.6 (Figure 1).

**Figure 2:**
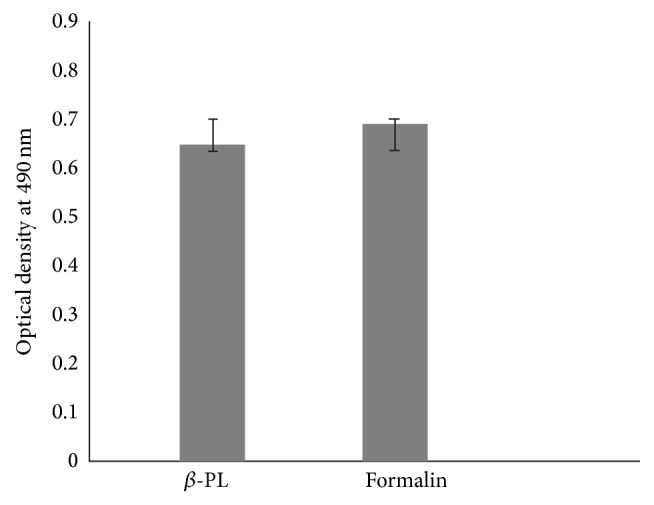
Antigenicity of *β*-PL and formalin inactivated WN preparations evaluated by indirect ELISA.

Antigenicity of formalin and *β*-PL inactivated virus preparation was checked by indirect ELISA. No significant difference in antigenicity of the virus inactivated by the two inactivating agents was observed (Figure 2).

### 3.3. Cell Toxicity Evaluation by MTT Assay

Cytotoxicity of inactivated WNV by both *β*-PL and formalin inactivation on BHK-21 cell lines was quantitatively determined by MTT assay. It was observed that the formalin treated virus induced cell death in 13% of cells and the *β*-PL treated virus induced cell death in 12% of cells.

### 3.4. Humoral Immune Response

**Figure 3:**
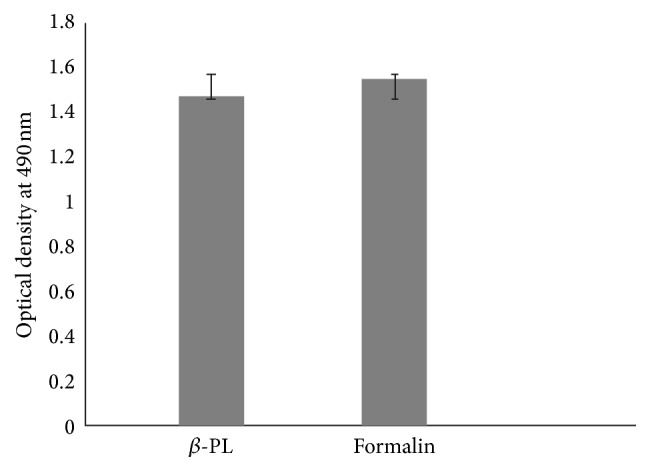
Humoral immune response induced in mice model by *β*-PL and formalin inactivated WN preparations evaluated by indirect ELISA.

Humoral antibody response in postimmunized mice sera measured by indirect ELISA showed anti-WNV antibody titre moderately higher in formalin inactivated antigen compared to *β*-PL inactivated antigen. Statistical analysis between the two groups showed no significant difference in induction of humoral immune response (Figure 3). ELISA titer of JEV specific polyclonal sera is negligible and comes into cut-off of negative value.

The neutralizing antibody response of the antigen obtained by inactivation by either of the methods (*β*-PL or Formalin) showed higher PRNT_50_ titers (*P* < 0.02) as compared to the control mice group. Although both the experimental groups showed no significant differences in titer levels, but both the inactivation procedures elicited equal protective efficacy. However, the postimmunized WNV specific mice sera showed cross protective neutralizing antibodies of 1 : 25 against JEV.

## 4. Discussion

WNV invasion continues to expand its geographic distribution. Therefore, studies on safety and efficacy of different vaccine preparation methods are important to develop intervention strategies. The main objective of our study was to evaluate and compare the efficiency of two virus inactivating agents, formalin and *β*-PL, on WNV. The alkylating agents, formalin and *β*-PL inactivate viruses* via* chemical reaction with viral capsid proteins and nucleic acids. They were tested in separate experiments at standard usage concentrations [17, 18].

The result obtained for *β*-PL and formaldehyde inactivation demonstrated that complete inactivation was achieved within 48 to 120 hrs in media containing 0.1%  *β*-PL and 0.2% formaldehyde, respectively. The amount of *β*-PL required to inactivate the virus differs depending upon the groups of the virus. For arbovirus groups, 0.05 to 0.1%  *β*-PL is sufficient [17]. Formalin was used for inactivation of a hepatitis A virus, and the vaccine was found to be safe and immunogenic in experimental models [18]. It was observed that for formalin inactivation; concentration, pH, and medium composition were critical. A doubled concentration of formalin at 26°C, pH 8.4, for 48 hours was sufficient for inactivation [24]. An incomplete inactivation procedure may prove to be fatal for public health. Outbreak of Venezuelan equine encephalitis was a result of incomplete inactivation of the vaccines prepared by formalin inactivation [25]. *β*-PL causes structural modification by alkylation and depurination of nucleic acid and is capable of inactivating viruses in 10–15 min at 37°C whereas formalin requires at least 24–96 hrs at 4°C–37°C conditions for inactivation under similar conditions [26, 27]. Although the exact mechanism of RNA degradation through formalin inactivation is not known, it may be due to formalin reaction with viral RNA, forming N-methylol (N-CH2OH), followed by an electrophilic attack to form a methylene bridge between amino groups resulting in cross-linkage between nucleic acids and proteins. This cross-linking inhibits reverse transcription of the extracted RNA and interferes in cDNA synthesis [28].

Studies demonstrated significantly higher degree of antigenicity with *β*-PL inactivated virus vaccines [29]. In a study comparing *β*-PL and formalin inactivaton of poliovirus, significant higher antigen recoveries were found in *β*-PL inactivated antigen compared to formalin antigen [30]. However, in our study, no significant differences were observed in antigenic potential between the *β*-PL and formalin inactivated WNV. Both showed equal antigenic potency as measured by indirect ELISA using monoclonal antibody against* Flavivirus* (HX-B). Moreover, it may also be supported by high antigen titer obtained in HA assay in both the inactivation processes. In *β*-PL inactivation, highest titer was obtained with pH 6.6, whereas formalin inactivation showed a high titer with VADs Ph 6.2, 6.6, and 6.8. The pH of the diluent had a critical influence on the HA reaction, affecting not only the titer of the HA preparation but also the appearance of the erythrocyte agglutination pattern. It may so happen that, during *β*-PL hydrolysis, the pH decreases, resulting in a conformational change in HA as it has been observed in case of influenza virus [31].

Studies carried out in humans and in animal model have indicated the importance of an effective humoral response in preventing* Flavivirus* infection both in the periphery and within the central nervous system [32, 33]. In the present study, both the formaldehyde and *β*-PL inactivation processes were found to be equally efficient for inactivation of WNV and were able to elicit humoral immune response against WNV. However, the present study is limited by showing the humoral immune response only till 7 days post inoculation (PI). Studies must be done to screen for immunogenic response every 3 months PI for 1 year. Moreover, other inactivating agents like binary ethylenimine [34] should be further compared and studies assessing influence of different inactivation agent on innate immunity (cell mediated immune response) should be done to formulate the best suitable method for WNV vaccine preparation.
